# A Case of Right Middle Lobe Syndrome: A Rare Diagnosis Behind Chronic Pulmonary Symptoms

**DOI:** 10.7759/cureus.95099

**Published:** 2025-10-21

**Authors:** Erum Zahid, Nisha K Sapkota, Aditi Parulkar, Anusha Akella, Anup Shrestha, Minhaz Murshad, Tutul Chowdhury

**Affiliations:** 1 Pulmonary and Sleep Medicine, Brookdale University Hospital Medical Center, Brooklyn, USA; 2 Medicine, Interfaith Medical Center, Brooklyn, USA; 3 Internal Medicine, One Brooklyn Health, Brooklyn, USA; 4 Internal Medicine, Interfaith Medical Center, Brooklyn, USA; 5 Pulmonary and Critical Care Medicine, One Brooklyn Health, Brooklyn, USA; 6 Hospital Medicine, Avera McKennan Hospital and University Health Center, Sioux Falls, USA; 7 Internal Medicine, Jersey Shore University Medical Center, Neptune, USA

**Keywords:** atelectasis, bronchoscopy, chronic cough, right middle lobe syndrome, streptococcus pneumoniae

## Abstract

Right middle lobe syndrome (RMLS) is an uncommon pulmonary condition characterized by recurrent or chronic atelectasis or infection of the right middle lobe, often due to either obstructive or non-obstructive mechanisms. We present the case of a 62-year-old male with a history of chronic obstructive pulmonary disease (COPD), prediabetes, and prior tobacco use, who presented with mid-sternal chest pain and a chronic dry cough. Imaging revealed right middle lobe collapse without endobronchial obstruction on bronchoscopy. A mucus plug was identified and removed, revealing edematous mucosa but no visible lesions. Cultures grew *Streptococcus pneumoniae* sensitive to ceftriaxone, while acid-fast bacilli (AFB) and fungal cultures were negative. Pathology demonstrated mild chronic inflammation with no malignancy. The patient responded well to antibiotics and inhaler therapy, with radiologic evidence of partial resolution on follow-up imaging. This case highlights the diagnostic challenges of RMLS in adults and underscores the importance of considering this rare entity in patients with chronic pulmonary symptoms and localized lobe collapse. Early bronchoscopy and appropriate treatment can lead to clinical and radiologic improvement.

## Introduction

Right middle lobe syndrome (RMLS) is a relatively uncommon clinical entity involving right middle lobe opacification; it generally refers to atelectasis in the right middle lobe of the lung. It was first identified in the 1940s by Graham et al. in a case series involving 12 patients with non-tuberculous atelectasis [1]. RMLS lacks a universally accepted definition, but it is generally characterized by recurrent or chronic middle lobe atelectasis or infection, which occurs through either of these two main pathophysiological mechanisms: obstructive or non-obstructive. Due to its rarity, the incidence and prevalence of RMLS remain poorly defined. However, it is more commonly reported in pediatric asthmatic patients [2], and some studies suggest a higher prevalence in females than males. There is very little epidemiological information about middle lobe syndrome (MLS). Numerous case series and national investigations have shown that the female-to-male ratio is larger and that women tend to present later in life than males. Furthermore, the most prevalent etiology of MLS, which accounts for more than 60% of reported cases, is the poorly understood nonobstructive form brought on by persistent inflammation [3,4]. Underlying lung diseases such as chronic obstructive pulmonary disease (COPD) may predispose patients to RMLS due to mucus plugging and impaired mucociliary clearance [3]. The clinical presentation of RMLS varies widely, ranging from asymptomatic to a patient experiencing chronic cough, dyspnea, chest discomfort, wheezing, and recurrent respiratory infections. Radiographic findings include atelectasis, bronchiectasis, or pulmonary infiltrates [4]. Given the nonspecific symptoms and potential for misdiagnosis, timely recognition and intervention are crucial for optimal patient outcomes. This case report describes a 62-year-old male with a history of recurrent respiratory tract infections, ultimately diagnosed with RMLS. By highlighting this case, we aim to emphasize the importance of considering RMLS in patients with persistent pulmonary symptoms, facilitating timely diagnosis and appropriate management.

## Case presentation

We present a case of a 62-year-old male with a past medical history of COPD, a former smoker, hepatic cavernous hemangiomas, and prediabetes. He presented with mid-sternal chest pain, reproducible, associated with a chronic dry cough. The patient also complained of dyspnea on exertion. On evaluation, he had wheezing and decreased breath sounds on the right airfield; SaO2 was 98% on room air. Labs on admission were unremarkable (Table 1). 

**Table 1:** Laboratory investigation

Investigation	Value	Reference range
Hemoglobin	14.1	11.0-15.0 g/dL
Hematocrit	43.9	35-46%
White blood cell	5.5	3.8-5.3 10 × 6/uL
Platelets	230	130-400 10 × 3/uL
Glucose	114	80-115 mg/dL
Blood urea nitrogen	9	9.8-20.1 mg/dL
Creatinine	0.8	0.57-1.11 mg/dL
Sodium	143	136-145 mmol/L
Potassium	4.2	3.5-5.1 mmol/L
Chloride	104	98-107 mmol/L
Bicarbonate	30	23-31 mmol/L
Calcium	9.2	8.8-10.0 mg/dL
Albumin	4.2	3.2-4.6 g/dL
Magnesium	1.9	1.6-2.6 mg/dL
Brain natriuretic peptide (BNP)	22	10.0-100.0 pg/mL
COVID polymerase chain reaction (PCR)	Negative	Negative
High-sensitivity troponin I	<5	0.0-17.0 ng/L
Prothrombin time	11.1	9.8-13.4 seconds
International normalized ratio (INR)	1.01	0.85-1.15
Partial thromboplastin time (PTT)	34	24.9-35.9 seconds
Thyroid-stimulating hormone (TSH)	0.397	0.465-4.680 uIU/mL
Thyroxine (T4)	0.8	0.78-2.19 ng/dL

**Figure 1:**
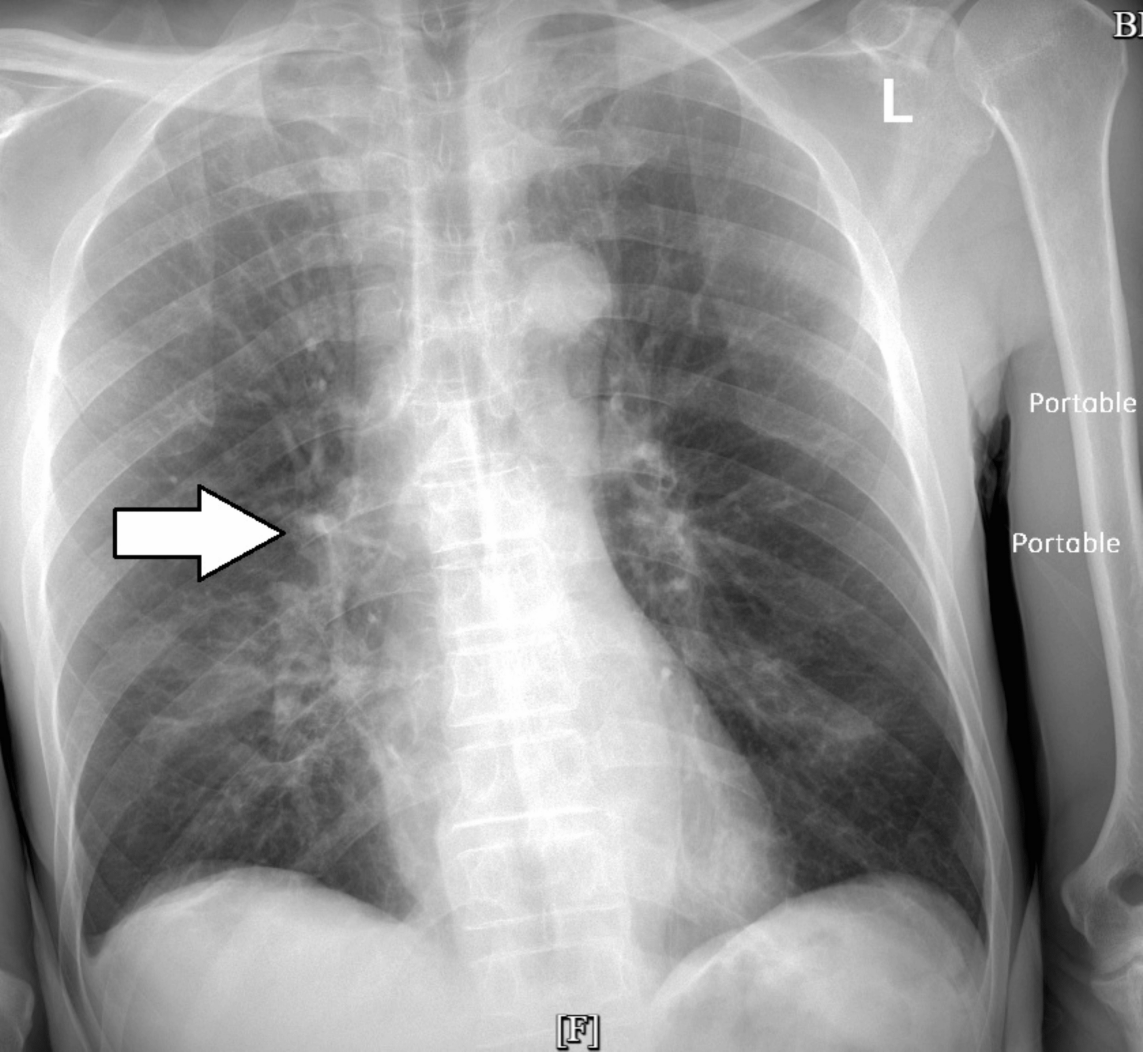
Chest X-ray showing patchy airspace opacity in the perihilar right lung (white arrow)

Chest X-ray revealed patchy airspace opacity in the perihilar right lung (Figure 1). 

**Figure 2:**
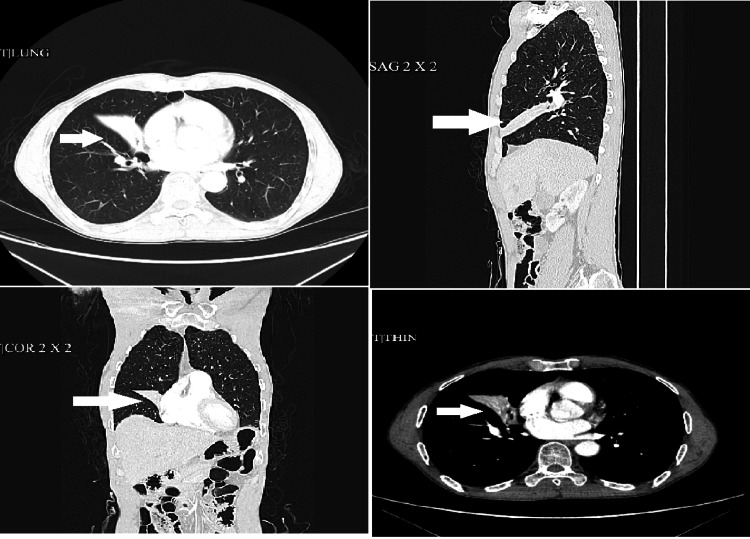
CT scan of the lung, total collapse of the right middle lobe

Computed tomography scan of the chest displayed collapse of the right middle lobe (Figure 2). The remaining lungs, mediastinum, and pleura were clear.

In the echocardiogram, left ventricular diastolic function was normal with normal left ventricular wall thickness and normal segmental wall motion.

**Figure 3:**
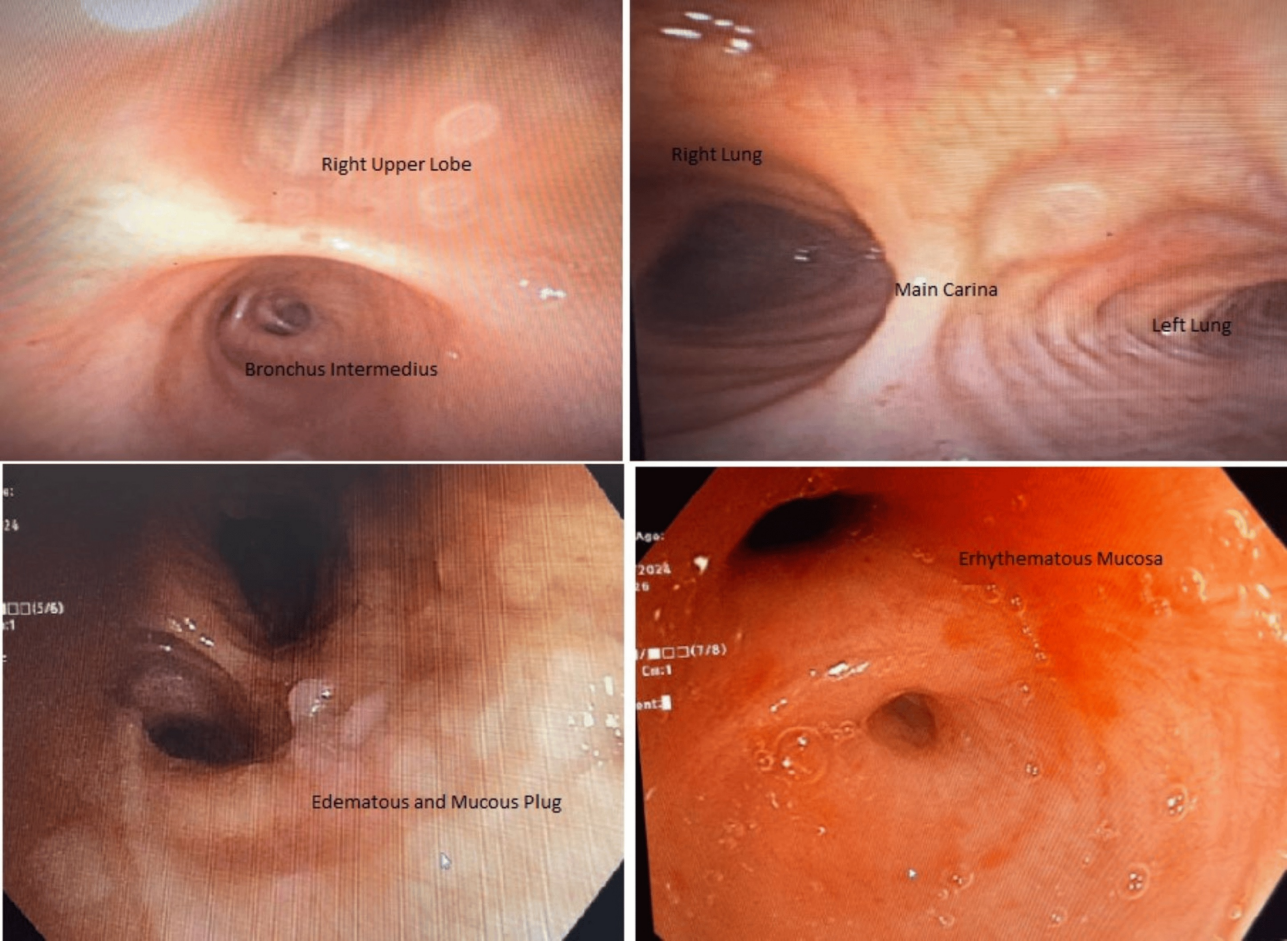
Bronchoscopy showing the right lung segments as depicted

Pulmonology was consulted due to the unclear etiology of the collapse. Bronchoscopy on the patient indicated edematous mucosa, a mucus plug with a narrowed right middle lobe bronchus. No endobronchial lesion identified, mucus plug removed, biopsies sent for pathological evaluation (Figure 3).

**Figure 4:**
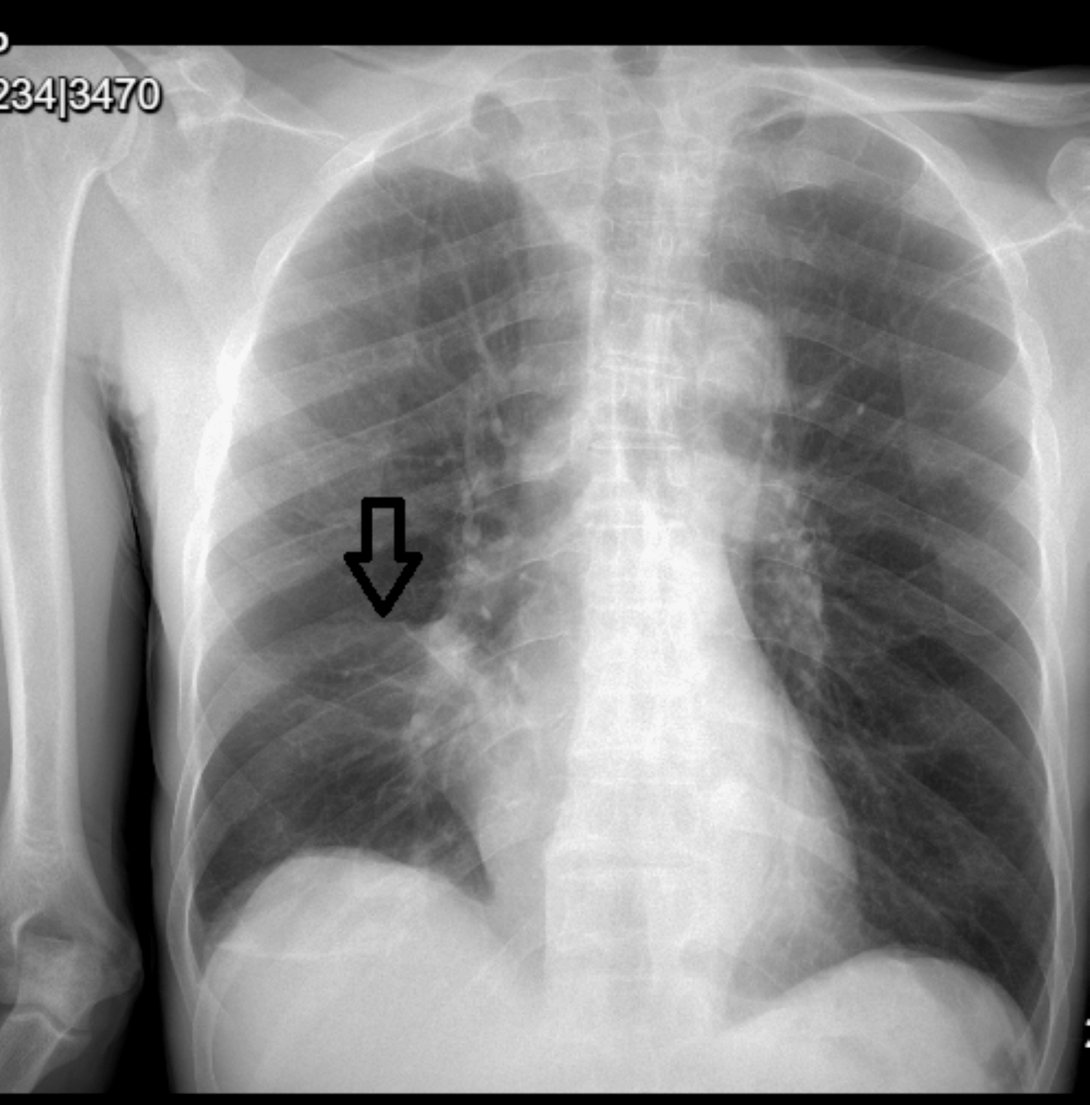
Chest X-ray showing partial atelectasis of the right middle lobe

The bronchial culture was positive for *Streptococcus pneumoniae,* susceptible to ceftriaxone. Acid-fast bacilli and fungal culture were negative. White blood cell count was 6.5. Repeat chest X-ray showed patchy airspace opacity in the perihilar right lung consistent with pneumonia with persistent partial atelectasis of the right middle lobe (Figure 4). Subsequent X-ray showed interval improvement. The patient was treated with IV ceftriaxone followed by oral amoxicillin. Biopsy from the right middle lobe showed benign bronchial gland hyperplasia and smooth muscle hypertrophy associated with mixed subacute and chronic inflammation. Cytology reports from right middle lobe brushing and bronchoalveolar lavage (BAL) indicated mild mixed subacute and chronic inflammation without any malignant cells. The patient was discharged in a stable condition with oral antibiotics and inhalers; he did not meet the criteria for home oxygen. Previous chest imaging from previous admissions showed similar characteristics.

**Figure 5:**
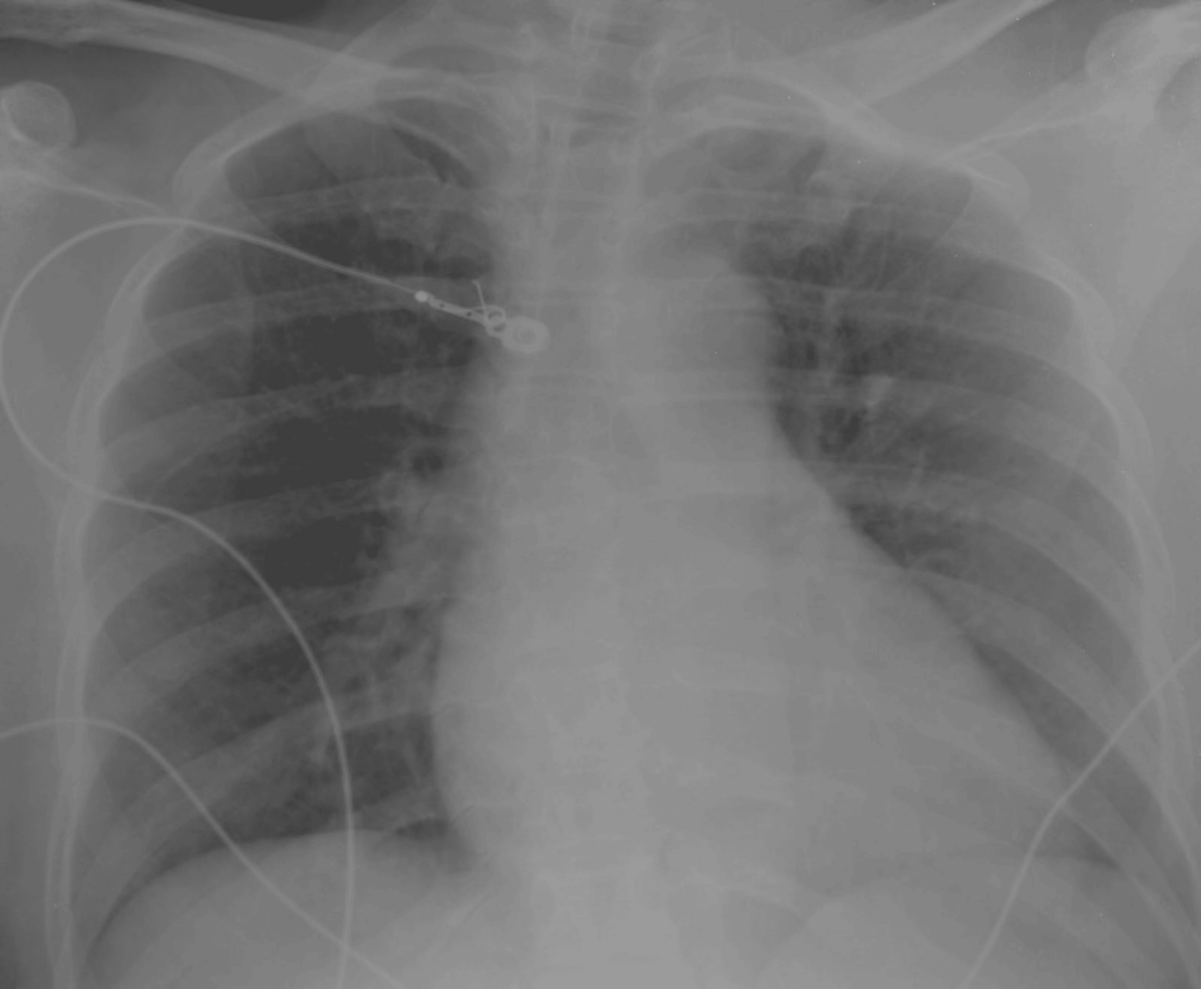
Normal chest X-ray

On a follow-up visit two months later, he reported symptomatic improvement. The follow-up chest X-ray was normal (Figure 5).

## Discussion

MLS is a relatively uncommon condition that affects the right middle lobe and/or left lingula of the lung. RMLS is a clinical entity characterized by recurrent or persistent collapse of the right middle lobe, typically resulting from a combination of intrinsic bronchial abnormalities and extrinsic factors such as lymph node enlargement or chronic inflammation [1]. The unique anatomy of the right middle lobe with its long, narrow bronchus and limited collateral ventilation predisposes it to atelectasis and recurrent infections [1,2]. In our case, the patient presented with a history of chronic dry cough without any overt signs of infection.

Despite its clinical importance, MLS lacks extensive epidemiological data, with only a few case series and nationwide studies available. Research has indicated a higher female-to-male ratio in MLS patients, with women generally presenting at a later age than men [3,4]. The pathophysiology of RMLS is multifactorial, involving both anatomical predisposition and acquired factors like persistent infections and inflammatory processes. It is recognized to involve two distinct pathophysiological pathways leading to recurrent middle lobe atelectasis, obstructive and non-obstructive [1]. Obstructive MLS is caused by extrinsic compression of the right middle lobe bronchus from lymphadenopathy or tumors like hamartomas, lung cancers, or metastases. Intraluminal obstructions can also result from conditions like sarcoidosis or mucus plugs [1,5]. Non-obstructive MLS occurs without visible obstruction and is linked to ineffective collateral ventilation, often due to chronic inflammation, infections, or bronchiectasis (Table 2) [1,5].

**Table 2:** Comparison between obstructive and non-obstructive right middle lobe syndrome MLS: middle lobe syndrome; RML: right middle lobe

	Obstructive right MLS	Non-obstructive right MLS
Causes	Endobronchial lesion (e.g., tumor, foreign body, mucus plug)	Inflammation, infection, or external compression impairing ventilation
Common etiologies	Bronchial tumor; foreign body; broncholith; lymph node compression	Chronic inflammation; asthma; post-infectious changes; allergic bronchopulmonary aspergillosis (ABPA)
Pathophysiology	Blocked airway → air resorption → lobe collapse	Impaired ventilation/perfusion mismatch due to inflammation or bronchial narrowing
Onset	Sudden or subacute	Gradual and recurrent
Symptoms	Cough; dyspnea; fever (if infected); hemoptysis (if tumor)	Chronic cough; recurrent infections; mild dyspnea
Radiologic findings	Complete or partial collapse of RML, possibly with mass or foreign body seen on CT	Collapse or volume loss of RML, tree-in-bud appearance, bronchiectasis
Treatment	Removal of obstruction (surgery, bronchoscopy), antibiotics if infected	Anti-inflammatory treatment, bronchodilators, chest physiotherapy
Bronchoscopy	Usually reveals a visible obstruction in the bronchus	Bronchial inflammation or narrowing, no visible blockage
Prognosis	Depends on cause - good if benign and treated early	Chronic course, often recurrent, but manageable

MLS has been associated with various microorganisms, including *Aspergillus*, *Histoplasma*, *Blastomyces*, *Streptococcus pneumoniae*, *Staphylococcus aureus*, and *Mycobacterium* species, among others [6,7]. Mucus plugging and chronic inflammation further contribute to airway obstruction, creating a cycle of recurrent infection and progressive lung damage.

The symptoms of MLS typically develop gradually. According to previous studies, the main symptoms include chronic cough, purulent sputum, unintended weight loss, fever, fatigue, hemoptysis, chest pain, and night sweats, and features consistent with recurrent pneumonia [2,7]. Diagnosis of RMLS relies on clinical suspicion, chest imaging, and bronchoscopy to rule out endobronchial lesions or foreign bodies [1]. High-resolution CT (HRCT) helps identify structural abnormalities, while pulmonary function tests may show obstructive or restrictive patterns based on disease chronicity. Initial diagnosis often involves posteroanterior (PA) and lateral chest X-rays, with the lateral view revealing a triangular density suggesting right middle lobe collapse [2]. HRCT provides detailed images of bronchial abnormalities, parenchymal changes, and lymph node enlargement. Flexible bronchoscopy assesses bronchial patency, identifies tumors, and obtains diagnostic samples, with histological findings typically showing bronchiectasis and foreign body reactions [6]. 

Treatment for MLS syndrome is directed at addressing its underlying cause [1,8]. In infectious cases, targeted antibiotic therapy is essential. For instance, MAC infections are managed with a regimen of macrolide, ethambutol, and rifampicin administered three times weekly for 12 months [9]. Adjunctive treatments such as bronchodilators, muco-lytics, and postural drainage help improve airway patency and facilitate mucus clearance [10]. Several studies report a favorable prognosis with proper treatment, although outcomes vary based on disease severity and complications [8]. However, in severe or medication-resistant cases, surgical intervention may be necessary. Thoracoscopic resection of the middle lobe has emerged as a safe and promising minimally invasive option after thorough preoperative evaluation, while lobectomy of the right middle lobe remains a viable alternative for persistent cases [9,10].

This case underscores the importance of a multidisciplinary approach in both diagnosing and managing RMLS, integrating radiologic, bronchoscopic, and medical therapies. Early and accurate diagnosis, combined with individualized treatment strategies, is essential to prevent chronic pulmonary complications and enhance long-term outcomes. Ongoing research into the optimal diagnostic and therapeutic strategies continues to refine our understanding and management of this challenging condition.

## Conclusions

This case highlights the importance of considering RMLS in patients with persistent pulmonary symptoms, particularly those with underlying lung disease. RMLS is an underrecognized condition that often mimics pneumonia or malignancy, leading to unnecessary diagnostic procedures and diagnostic delays. Increased awareness among pulmonologists and physicians is essential for timely identification and appropriate management. Bronchoscopy plays a crucial role as both a diagnostic and a therapeutic tool in these cases. Further research is needed to establish optimal long-term management strategies and to better understand the association between RMLS and underlying lung conditions such as asthma and COPD.
